# Analysis of Neurophysiological Correlates of Mental Fatigue in Both Monotonous and Demanding Driving Conditions

**DOI:** 10.3390/brainsci15091001

**Published:** 2025-09-16

**Authors:** Francesca Dello Iacono, Luca Guinti, Marianna Cecchetti, Andrea Giorgi, Dario Rossi, Vincenzo Ronca, Alessia Vozzi, Rossella Capotorto, Fabio Babiloni, Pietro Aricò, Gianluca Borghini, Marteyn Van Gasteren, Javier Melus, Manuel Picardi, Gianluca Di Flumeri

**Affiliations:** 1 Department of Computer, Automatic and Management Engineering, Faculty of Information Engineering, Computer Science and Statistics, Sapienza University of Rome, 00185 Rome, Italy; cecchetti.1844914@studenti.uniroma1.it (M.C.); vincenzo.ronca@uniroma1.it (V.R.); pietro.arico@uniroma1.it (P.A.); 2 Department of Molecular Medicine, Faculty of Pharmacy and Medicine, Sapienza University of Rome, 00185 Rome, Italy; guinti.1747302@studenti.uniroma1.it (L.G.); andrea.giorgi@uniroma1.it (A.G.); dario.rossi@uniroma1.it (D.R.); gianluca.borghini@uniroma1.it (G.B.); 3 BrainSigns srl, Via Tirso, 14, 00198 Rome, Italy; alessia.vozzi@brainsigns.com (A.V.); fabio.babiloni@uniroma1.it (F.B.); 4 Department of Anatomical, Histological, Forensic and Orthopedic Sciences, Sapienza University of Rome, 00161 Rome, Italy; rossella.capotorto@uniroma1.it; 5 Department of Physiology and Pharmacology, Faculty of Pharmacy and Medicine, Sapienza University of Rome, 00185 Rome, Italy; 6 College of Computer Science and Technology, Hangzhou Dianzi University, Hangzhou 310018, China; 7 Instituto Tecnológico de Castilla y León (ITCL), 09001 Burgos, Spainjavier.melus@itcl.es (J.M.); 8 European Driving School Association, 1040 Brussels, Belgium; manuel.picardi@efa-eu.com

**Keywords:** road safety, simulated driving, mental fatigue, multimodal assessment, EEG index

## Abstract

**Background/Objectives**: Mental fatigue during driving, whether passive (arising from monotony) or active (caused by cognitive overload), is a critical factor for road safety. Despite the growing interest in monitoring techniques based on neurophysiological signals, current biomarkers are primarily validated only for detecting passive mental fatigue under monotonous conditions. The objective of this study is to evaluate the sensitivity of the MDrow index, which is based on EEG Alpha band activity, previously validated for detecting passive mental fatigue, with respect to active mental fatigue, i.e., the mental fatigue occurring in cognitively demanding driving scenarios. **Methods**: A simulated experimental protocol was developed featuring three driving scenarios with increasing complexity: monotonous, urban, and urban with dual tasks. Nineteen participants took part in the experiment, during which electroencephalogram (EEG), photoplethysmogram (PPG), and electrodermal activity (EDA) data were collected in addition to subjective assessments, namely the Karolinska Sleepiness Scale (KSS) and the Driving Activity Load Index (DALI) questionnaires. **Results**:The findings indicate that MDrow shows sensitivity to both passive and active mental fatigue (*p* < 0.001), thereby demonstrating stability even in the presence of additional cognitive demands. Furthermore, Heart Rate (HR) and Heart Rate Variability (HRV) increased significantly during the execution of more complex tasks, thereby suggesting a heightened response to mental workload in comparison to mental fatigue alone. Conversely, electrodermal measures evidenced no sensitivity to mental fatigue-related changes. **Conclusions**: These findings confirm the MDrow index’s validity as an objective and continuous marker of mental fatigue, even under cognitively demanding conditions.

## 1. Introduction

Mental fatigue during driving is a significant contributing factor to road safety issues, accounting for 20% of fatal accidents [1], with consequences that are analogous to those associated with alcohol consumption [2]. This is because, although driving is a daily routine for many, it is a multi-faceted task that involves all the basic human psychological processes and whose demands can vary significantly. Depending on the context, it can range from monotonous and repetitive to highly demanding, requiring different levels of mental effort that can gradually lead to mental fatigue [3]. To describe better the different ways in which mental fatigue can manifest, the literature distinguishes two main forms of mental fatigue in driving. Passive mental fatigue occurs when the driver operates in low-stimulation conditions, such as monotonous driving or on highly predictable road segments, where the lack of external stimuli leads to progressive cognitive disengagement [4]. In contrast, active mental fatigue arises in high-complexity environments, such as urban settings, where the driver is exposed to elevated cognitive demands—for instance, managing heavy traffic, frequent maneuvers, or secondary tasks, which may lead to cognitive overload [4]. More generally, mental fatigue during driving can be classified into two primary categories: sleep-related (SR) and task-related (TR) fatigue. TR fatigue arises from the cognitive demands of the task being performed and is the one that can be further divided into active and passive forms [5]. SR fatigue, on the other hand, is linked to circadian rhythms and sleep quality, with well-documented reductions in vigilance occurring between 02:00 and 06:00 and between 14:00 and 16:00, as well as under conditions of sleep restriction or deprivation. Overall, mental fatigue in driving can be shaped by both external environmental factors and internal physiological processes [6]. Once mental fatigue sets in, it has been shown to cause deterioration in cognitive performance, including reduced vigilance, increased reaction time, and impaired decision-making capacity, thereby compromising safety [7,8].

As a result, the detection of driver mental fatigue represents a crucial open challenge that has gained increasing attention in the field of driver monitoring. To address this issue, several techniques have been developed to detect driver mental fatigue, some of which have been implemented in the Advanced Driver Assistance Systems (ADAS) [9]. These systems are generally classified into two categories: (I) monitoring vehicle-based systems and (II) monitoring driver-based systems. However, both approaches present notable limitations. They are unable to act before the sudden onset of drowsiness, often suffer from low accuracy and high false positive rates, and do not account for inter-individual variability [10]. Mental fatigue does not always manifest in the same way in vehicle dynamics or driver behavior [11].

In order to overcome these limitations, research has played a central role by investigating mental fatigue dynamics through physiological and neurophysiological signals, including electroencephalogram (EEG), electrooculogram (EOG), heart activity, and skin sweating, which are collected directly from the driver and which offer a more objective, sensitive, and timely way to assess the internal state of the individual, and can therefore provide a solid scientific foundation for the development of monitoring techniques that are reliable, early-detecting, and customizable [12,13]. These techniques, based on monitoring neurophysiological changes, could be implemented within advanced monitoring systems. Among these, those based on EEG represent the most promising candidates: indeed, EEG has the highest temporal resolution among neurophysiological signals (in the millisecond range) [14], indirectly captures brain activity, and has been found to be particularly effective in assessing drivers’ cognitive state. For this reason, it is widely recognized as the gold standard for measuring mental fatigue in controlled experimental settings. However, both laboratory and wearable EEG systems, as well as those used for EOG, which in many cases are integrated within the same device, are often invasive, and this could interfere with the driving task itself, putting drivers at risk. Alternatively, they could induce a sense of discomfort, which would in turn alter the natural induction, perception, and manifestation of mental fatigue while driving. On the other hand, biosignals like heart activity or skin sweating can now be easily collected through wearable devices like smartwatches or wristbands. However, these signals are less accurate than EEG in intercepting changes in mental states. An optimal approach would be to use the EEG as a ground truth to label and study other biosignals, which can be comfortably and seamlessly collected using wearable devices. To make this possible, defining an objective neurophysiological index of mental fatigue is a key priority [15], which can serve as a reference for the calibration and validation of these multimodal solutions.

In this context, EEG has been widely studied for its potential in mental fatigue monitoring. Specifically, low-frequency oscillations are widely recognized as reliable markers of mental fatigue or increasing drowsiness [16,17]. Several studies have examined Alpha band activity, in which synchronization phenomena, especially over parietal sites, named “Alpha spindles/bursts”, have been highlighted as very promising biomarkers [12,18,19,20], while others have investigated Theta and Delta bands [16,21,22,23]. These studies have all confirmed an increase in slow oscillations under mental fatigue conditions.

Based on these findings, the Mental Drowsiness (MDrow) Index was recently introduced by Di Flumeri and colleagues [20] to detect mental drowsiness through transient episodes of Alpha wave synchronization analysis in parietal regions. The index is based on Global Field Power (GFP) in the Alpha band, normalized to the maximum value recorded during the eyes-closed resting state (EC). Because activity during the eyes-closed resting state reflects reduced sensory engagement, and mental drowsiness is characterized by decreased alertness, together with reduced sensory and cognitive processing, the rationale for the MDrow index is that the greater the similarity between brain activity during task performance and during eyes-closed rest, the higher the likelihood that the subject is experiencing a state of mental drowsiness. The so-defined MDrow index has been demonstrated to be positively correlated with mental fatigue levels. As previously mentioned, even if EEG represents an ideal candidate to intercept the onset of mental fatigue in an operative context, its adoption in real-world settings is hindered by its invasiveness. For this reason, researchers have largely investigated the possibility to characterize the fatigued state using other biosignals, such as electrooculography (EOG), photopletismography (PPG), and electrocardiographic (ECG) and electrodermal activity (EDA). A previous study demonstrated that EOG might be used to compute key features that can be used to characterize a mentally fatigued state. In particular, both eye blink rate (EBR) and eye blink duration (EBD) have been repeatedly found to increase with growing mental fatigue, making them reliable indicators in various operational settings [24,25]. In addition to ocular signals, heart activity, typically measured via PPG and ECG, has also proven useful in tracking mental fatigue-related autonomic fluctuations. Heart rate variability (HRV), shaped by the balance between sympathetic and parasympathetic influences, tends to shift during mental fatigue episodes. Specifically, the LF/HF ratio tends to increase due to elevated low-frequency power, indicating enhanced sympathetic activation [26,27]. Although explored to a lesser extent, electrodermal activity (EDA) has also been considered in this domain. Variations in skin conductance may signal shifts in arousal and autonomic state during mental fatigue, pointing to its potential utility in mental fatigue monitoring [28,29].

Although sensitive neurophysiological biomarkers of mental fatigue have been identified, such as MDrow [20], these are primarily derived from studies conducted in monotonous driving scenarios. These environments promote spontaneous mental fatigue [30], facilitate the isolation of mental fatigue correlates, and allow for strict experimental control due to their simplicity and lack of external interference. The MDrow index, along with other EEG-based biomarkers of mental fatigue, has primarily been validated in monotonous driving scenarios, which correspond to conditions of passive mental fatigue. Monotonous driving represents only a small subset of real-world situations. In contrast, daily driving is often far from monotonous, involving variable and dynamic scenarios that require multitasking and rapid decision-making. Therefore, these biomarkers, such as MDrow, have intrinsic limitations. In more cognitively demanding situations, EEG signal correlates may also reflect other cognitive processes, which compromises the index’s specificity. To validate an indicator for a real-world setting, it must be demonstrated that the underlying neurophysiological correlate remains stable and measurable regardless of task complexity.

Due to the importance of mental fatigue in driving and the inadequacy of existing tools for assessing it under realistic conditions, this study aims to address this research gap through the following steps:

(i) Developing an experimental protocol able to induce active and passive mental fatigue through driving scenarios with increasing cognitive complexity;
(ii) Assessing the sensitivity of the MDrow index, previously validated for passive mental fatigue, in detecting active mental fatigue under more cognitively demanding conditions;
(iii) Investigating how additional physiological parameters related to heart activity and skin sweating behave during periods of high MDrow activation to evaluate their potential as indicators of mental fatigue in active and passive contexts.

## 2. Materials and Methods

### 2.1. Participants

Nineteen (19) licensed drivers with normal or corrected-to-normal vision were recruited on a voluntary basis. Participants were selected in order to ensure a homogeneous sample of adults with ages ranging from 19 to 39 years (mean: 27.0 ± 4.7) and driving experience. The sample included 9 males and 10 females. All participants were in good health and had no history of neurological or psychiatric disorders, medication use, substance abuse, or symptoms of motion sickness. Each participant provided written informed consent after receiving a detailed explanation of the study. The experiment was conducted in accordance with the 2008 revision of the Declaration of Helsinki and was approved by the Ethics Committee of the University of Rome “La Sapienza.” Only aggregate results are reported to protect participant privacy.

### 2.2. Experimental Design and Protocol

The experiment was conducted after lunchtime (in our case, approximately between 1:30 p.m. and 3:30 p.m.), a point during daytime in which people more prone to the onset of mental fatigue because of circadian rhythms [31], and after the participant had their lunch. The simulation setup included a steering wheel, manual gearshift, and three screens for scenario visualization (Table 1). Driving environments were simulated using IRIS software (Instituto Tecnologico de Castilla y Leon, Burgos, Spain, https://itcl.es/en/research/simulation-and-immersive-technologies-area-itcl/, accessed on 15 September 2025). A tablet positioned next to the driver, similar to the traditional infotainment screens used inside the cars, was used to administer the secondary task while driving.

**Figure 1:**
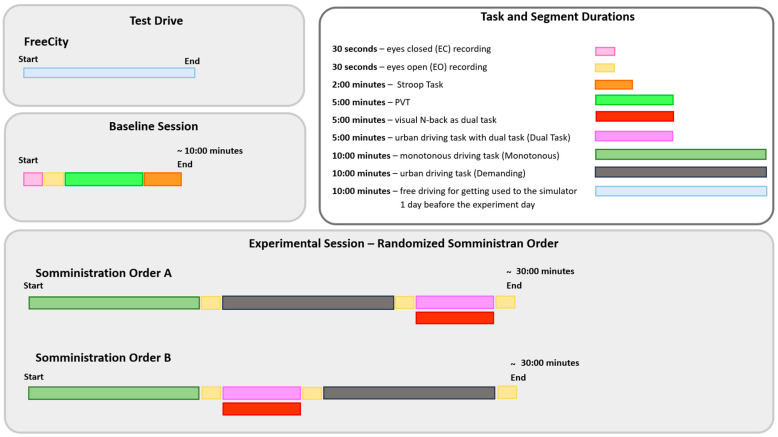
Overview of the experimental protocol and task structure. The top-left section shows the two preliminary phases: the Test Drive, carried out in the FreeCity environment to familiarize participants with the simulator 1 day before the experiment, and the Baseline Session, lasting approximately 10 min, which was performed immediately before the Experimental Session. This baseline included recordings during eyes-closed (EC) and eyes-open (EO) resting states, followed by three cognitive tasks: a Stroop task (2 min), a Psychomotor Vigilance Task (PVT) (5 min), and a visual N-back task (5 min). The top-right section summarizes the duration of all tasks using color-coded bars, including the three driving conditions: the monotonous driving task (10 min), which was low in environmental stimulation; the demanding driving task (10 min), featuring complex urban scenarios; and the dual-task driving condition (5 min), performed in the same environment as the demanding task but with the addition of a concurrent visual N-back task to increase cognitive load. The bottom section illustrates the two pseudo-randomized administration orders (A and B) adopted during the Experimental Session, each lasting approximately 30 min, and shows how the tasks were sequenced across participants to control for order effects.

To achieve the study objectives, a two-phase experimental structure was designed. The first phase, the Baseline Session, aimed to collect neurophysiological reference data. The second phase, the Experimental Session, was designed to induce and monitor mental fatigue through increasingly complex driving scenarios (Figure 1).

During the Baseline Session, participants underwent a series of neurophysiological recordings and cognitive tasks to establish a stable reference condition for each individual. Specifically, two short resting-state EEG recordings were conducted: one with the eyes closed (EC) and one with the eyes open (EO), each 30 s long. Participants then completed two tasks: a five-minute Psychomotor Vigilance Task (PVT) and a two-minute Stroop Task. These tasks were designed to collect baseline physiological data in the absence of mental fatigue.

The Experimental Session involved three simulated driving scenarios, each designed to evoke a different level of cognitive load, presented in a pseudo-randomized order (Table 1). The three driving tasks were as follows:

- Monotonous Driving (10 min): This task was conducted within a virtual environment reproducing the Roma’s Fair. Its internal road infrastructure is similar to an urban infrastructure, with 90-degree intersections, a zebra crossing, and few road signs, but the environment is very “monotonous”, basically consisting of the several hangars of the fair. In other words, it was a stimulus-free context (no traffic, pedestrians, or dynamic elements). Participants were instructed to maintain a constant speed of 40 km/h along a repetitive route, i.e., the external ring, almost 1.100 m long. This task was designed to induce passive mental fatigue.
- Demanding Driving (10 min): This task was conducted in a complex urban environment with vehicles, pedestrians, intersections, and road signs. Participants had to maintain a speed limit of 50 km/h and follow directions from a virtual navigation system. This condition was intended to induce active mental fatigue through the continuous processing of environmental information.
- Dual-Task Driving (5 min): This was conducted in the same environment as the Demanding Driving scenario, but it included a simultaneous secondary task (visual N-back − n = 1 − task [32]), in which the stimuli were pictures consistent with a urban driving scenario (cars, motorbikes, and pedestrians) proved to induce cognitive overload [33]. This condition aimed to test the robustness of neurophysiological mental fatigue indicators under multiple mental demands.

A summary table is provided below to visually illustrate the characteristics of each driving scenario, including a screenshot of the virtual environment, a schematic map of the route, and a description of the experimental setup.

The order of the two demanding driving tasks (demanding and dual-task) was randomized to distinguish between effects due to exposure duration (time on task) and those related to task-specific workload. Additional eyes-open (EO) EEG recordings were collected between each driving session to monitor the progression of mental fatigue over time in rest conditions.

### 2.3. Subjective Data Collection

In the present experimental protocol, subjective measures were used to complement the neurophysiological data by assessing participants’ perceived levels of mental fatigue and the mental workload related to each driving task. Two standardized questionnaires were selected for this study: the Karolinska Sleepiness Scale (KSS) [34] and the Driver Activity Load Index (DALI) [35].

The KSS was administered multiple times throughout the experiment (at baseline and after each driving task) to monitor perceived sleepiness over time. The DALI was administered at the end of each driving task to assess perceived cognitive workload based on task complexity.

### 2.4. Neurophysiological Data Collection and Processing

EEG signals were recorded using the Mindtooth Touch system (Brain Products GmbH, Gilching, Germany) at a sampling frequency of 125 Hz [36,37]. Eight water-based electrodes were placed over the frontal and parietal regions (AFz, AF3, AF4, AF7, AF8, Pz, P3, and P4) and referenced to the left mastoid with grounding to the right. Impedance was kept below 50 kΩ. The EEG signal was filtered using a fifth-order Butterworth filter (2–30 Hz) and cleaned of artifacts using a 50 Hz notch filter, a Wiener filter [38], and o-Clean method for correcting ocular blinks [39]. After this pre-processing, data segments still affected by artifacts were removed. Data were segmented into one-second epochs. Artifact rejection was performed using EEGLAB’s pop_eegthresh function, which automatically removed epochs with amplitude exceeding ±100 μV across EEG channels [40].

Then, the global field power (GFP) in the Alpha band was computed over the Parietal sites (P3, P4) for the analysis. The Alpha band was defined for each participant using their individual Alpha frequency (IAF) (mean: 9.3 ± 1.8 Hz), which was estimated during the EC baseline recording [41]. To isolate Alpha activity while minimizing interference from adjacent frequency bands, a strict Alpha band was defined as Alpha = (IAF − 1):(IAF + 1) Hz [42].

The GFP was calculated using a 1 s Hanning window over the parietal channels. Based on this, the MDrow index was computed as follows:

MDrow = Alpha_Parietal_ GFP [Task]/max(Alpha_Parietal_ GFP [Rest]), (1)

where “Rest” refers to the eyes-closed baseline recording (EC).

The MDrow index was calculated for the eyes-open (EO) recordings and driving tasks, which were segmented using 60 s windows with a 15 s overlap. The MDrow index was computed for each segment to track the temporal evolution of mental fatigue. This procedure was adopted to reduce transient fluctuations and highlight the underlying trend of mental fatigue, while maintaining adequate temporal resolution to follow its temporal evolution. The low mental fatigue (LowF) value was defined as the MDrow value during the first minute of monotonous driving, while the highest mental fatigue (HighF) value was defined as the maximum peak for each task. This resulted in ‘HighF—Monotonous’ for monotonous driving, ‘HighF—Demanding’ for demanding driving, and ‘HighF—Dual Task’ for dual-task driving.

Photoplethysmographic (PPG) signal was collected using the Shimmer3 GSR+ wearable device (Shimmer Sensing, Dublin, Ireland) at a sampling frequency of 64 Hz. The device was placed on the non-dominant wrist, with PPG sensor placed on the first finger to ensure high signal quality and comfortable wearability. The PPG signal was band-pass-filtered (fifth-order, 0.4–4 Hz) to remove the DC component and high-frequency noise while emphasizing the pulse component. Heartbeats were identified using a modified version of the Pan–Tompkins algorithm [43], and interbeat intervals (IBIs) were used to calculate heart rate (HR) and heart rate variability (HRV). HRV was estimated in the frequency domain by computing power spectral density (PSD) via the Lomb–Scargle periodogram, which provides an accurate analysis of unevenly spaced data. HRV was calculated in the low-frequency (LF: 0.04–0.15 Hz) and high-frequency (HF: 0.15–0.4 Hz) bands. The LF/HF ratio indicates autonomic balance.

Electrodermal activity (EDA) was acquired at 64 Hz using the Shimmer3 GSR+ device. A low-pass filter (1 Hz) was applied to eliminate high-frequency noise. The signal was processed using Ledalab [44,45] and analyzed via continuous decomposition analysis (CDA) [31], which allowed for the extraction of two main EDA components: skin conductance level (SCL, tonic) and skin conductance response (SCR, phasic).

Physiological parameters were calculated for all three driving tasks. The data were segmented into 60 s windows with 15 s overlaps. An analysis was performed of the time segments of both the PPG and EDA signals corresponding to maximum mental fatigue, as identified by the MDrow index, during the three driving tasks (monotonous, demanding, and dual-task). Physiological parameters were computed during these maximum mental fatigue windows and during the first minute of monotonous driving (LowF condition).

Heart rate (HR) and heart rate variability (HRV) values were normalized for each participant by dividing the value of each segment by the mean value recorded during the eyes-closed (EC) baseline, in order to account for individual physiological variability and to use a consistent resting condition as a reference. SCL and SCR values were normalized for each participant using their respective minimum and maximum values as follows:

SCL_norm_ = (SCL − SCL_min_)/(SCL_max_ − SCL_min_) SCR_norm_ = SCR/SCR_max_. (2)

### 2.5. Statistical Analysis

The Shapiro–Wilk test was preliminarily used to assess the normality of the data distributions, in order to determine if parametric or non-parametric statistics should be adopted. Multiple comparisons were analyzed using either repeated-measures ANOVA (parametric) or Friedman’s non-parametric ANOVA, depending on the results of the Shapiro–Wilk. When a statistically significant main effect arose from the ANOVA, paired comparisons were then performed using post hoc analysis. For all the tests, the statistical significance threshold was set at α = 0.05, corrected using the Holm method.

## 3. Results

This section was divided into different subparagraphs in order to organize all the results on the basis of the related data source, i.e., questionnaires (subjective results), and neurophysiological and performance data.

### 3.1. Subjective Results

**Figure 2:**
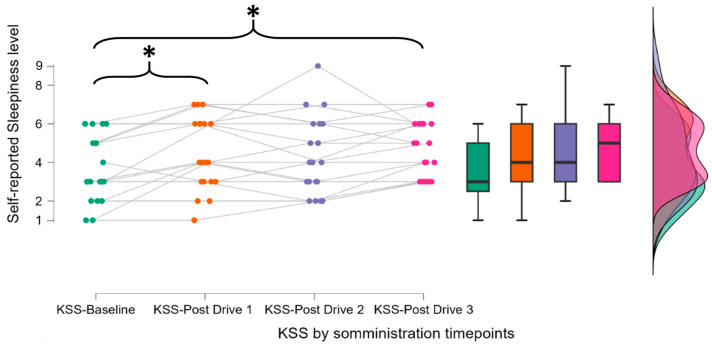
The participants reported a progressive increase in perceived mental fatigue over time. The average KSS score after the final task was around 5, indicating a state of “neither alert nor sleepy.” Both this moderate score and the significant differences observed after the monotonous drive and the last task confirm a gradual onset of mental fatigue. The asterisk (*) indicate statistically significant differences between the distributions connected by the corresponding brackets, as identified through post hoc comparisons following repeated-measures ANOVA. * indicates *p* < 0.05. Each color refers to the KSS score distribution at each evaluation point.

A preliminary analysis of the Karolinska Sleepiness Scale (KSS) scores was conducted to assess the effectiveness of the protocol in inducing mental fatigue. Due to the non-normal distribution of the data, the Friedman test was applied. The results revealed a significant effect of time (χ^2^ = 9.148, *p* = 0.027, η^2^ = 0.160), indicating a progressive increase in mental fatigue throughout the experiment (Figure 2).

Post hoc analysis with Holm’s correction showed significant differences between the baseline KSS value (KSS-Baseline) and subsequent scores, especially after the first driving task, the monotonous one, (KSS-Post Drive 1, *p* = 0.043) and final driving task (KSS-Post Drive 3, *p* = 0.047). This suggests that participants experienced progressive mental fatigue over time.

**Figure 3:**
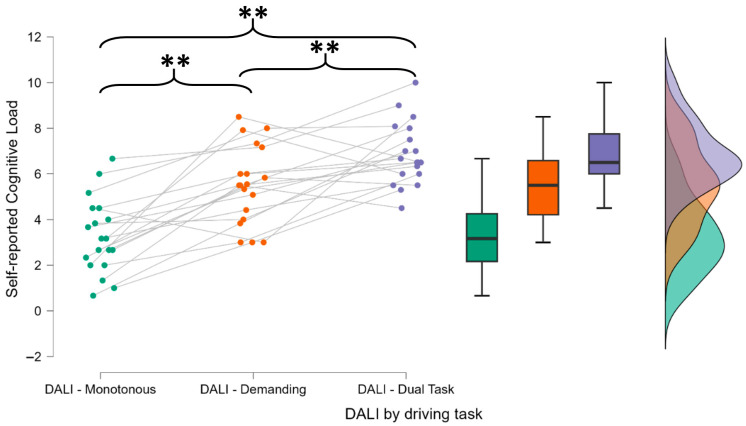
The participants reported significantly higher perceived cognitive load during the demanding driving tasks. These results suggest that, if mental fatigue arises in the more complex tasks, it could be attributed to an active mental fatigue condition caused by increased cognitive demands. The asterisks (*) indicate statistically significant differences between the distributions connected by the corresponding brackets, as identified through post hoc comparisons following repeated-measures ANOVA. ** indicates *p* < 0.01. Each color refers to the DALI score distribution for each driving condition.

Next, the DALI scale was analyzed to determine whether different driving conditions were associated with varying levels of perceived cognitive load. The Friedman test revealed a significant effect of driving condition (F = 45.128, *p* < 0.001), confirming that the tasks differed in cognitive demand (Figure 3).

Post hoc comparisons using the Holm correction indicated that the perceived mental workload was significantly higher in the demanding driving and dual-task conditions than in the monotonous driving condition (*p* < 0.01 for both comparisons). There was also a significant difference between the demanding driving and dual-task driving conditions (*p* < 0.01). These results support the hypothesis that complex simultaneous tasks increase the level of workload that is subjectively perceivable.

### 3.2. Performance Results

Following the results of the subjective measures, an analysis of participants’ performance in the N-back secondary task, performed simultaneously during the dual-task driving condition, was conducted to verify that they were subjected to an effective higher workload during the dual-task condition. The aim was to assess participants’ engagement throughout the task and to ensure that they consistently performed the secondary task while driving. To this end, we employed the Inverse Efficiency Score (IES) as the performance metric. In experimental cognitive psychology, performance is typically described by two dependent variables: the proportion of errors (PE) and the reaction time (RT) of correct responses. However, analyzing these measures separately can complicate interpretation, particularly in the presence of a speed–accuracy trade-off [46]. On average, accurate decisions require more time, while fast decisions are usually less accurate [47]. However, in some circumstances, both patterns can occur. For this reason, RT alone could have been misleading in our study, as longer latencies might indicate either disengagement from the task or simply the additional time needed to produce correct responses. The IES addresses this ambiguity by combining speed and accuracy into a single measure. It is computed as follows:

IES = RT/(1 − PE)(3)

where RT is the mean reaction time for correct responses and PE is the Proportion of Errors, i.e., the percentage of missed targets with respect to the total amount of target stimuli. The IES is expressed in seconds and reflects the effective time per correct response, providing a more robust estimate of sustained engagement.

For each participant, the IES was calculated at the first, third, and fifth minutes of the Dual-Task condition, corresponding to the beginning, middle, and end of the task. Since the data were not normally distributed (all *p* < 0.05), a Friedman test was applied. The results revealed no significant effect of time (χ^2^ = 0.889; df = 2, *p* = 0.641; Kendall’s W = 0.049), confirming that participants consistently continued performing the secondary task for the entire duration.

These results confirm that participants remained equally engaged throughout the secondary task, thereby validating that the dual-task condition was performed under a sustained higher cognitive load compared to the demanding driving condition.

### 3.3. Neurophysiological Results

**Figure 4:**
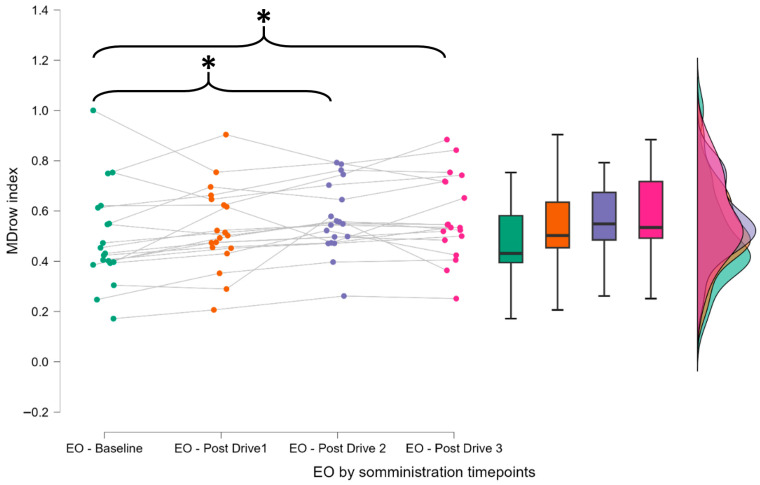
Progressive increase in the MDrow index across the experimental timeline during eyes-open (EO) resting-state conditions. This plot illustrates the variation in the MDrow index measured immediately after each driving task under EO conditions in which participants were not engaged in any active task. This design isolates the effect of mental fatigue, allowing for a more direct interpretation of neurophysiological changes. As shown in the graph, the MDrow index exhibited a progressive increase over time, supporting the objective validity of the experimental protocol by confirming that participants experienced a growing state of mental fatigue. The asterisk (*) indicate statistically significant differences between the distributions connected by the corresponding brackets, as revealed by post hoc comparisons following a repeated-measures ANOVA. * indicates *p* < 0.05. Each color represents the distribution of MDrow values recorded at the respective timepoints.

The EEG analysis focused on evaluating the MDrow index (Figure 4). In the first phase, the index was assessed under eyes-open (EO) conditions to verify its sensitivity towards mental fatigue over time. In fact, for each subjective assessment of mental fatigue, performed by filling the KSS, one EO condition was recorded; therefore, similar and coherent effects are expected when analyzing the MDrow index with respect to KSS scores (please refer to Figure 2). Repeated-measures ANOVA revealed a significant time effect (F = 6.489, *p* < 0.001). Post hoc comparisons using the Holm correction indicated that significant increases in the index were observed between the EO baseline and EO after the second driving task (*p* = 0.014) and between the EO baseline and EO after the third driving task (*p* = 0.021).

**Figure 5:**
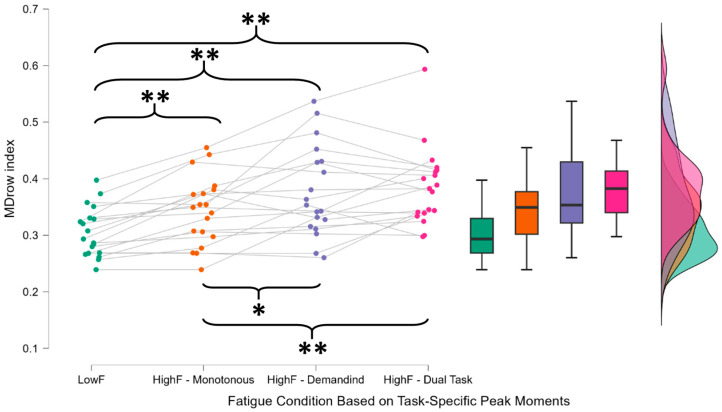
MDrow index across mental fatigue conditions identified through task-specific peak moments. For each participant, the EEG signal recorded during the three driving tasks (monotonous, demanding, and dual-task) was segmented using 60 s windows with a 15 s overlap, and the MDrow index was computed for each segment. The LowF value was defined as the MDrow index during the first minute of the monotonous driving task, assumed to represent the participant’s lowest mental fatigue level. The HighF value for each driving condition was defined as the maximum MDrow value across all segments within that task, calculated individually for each participant. This means that HighF values refer to different time windows across participants, with each corresponding to their peak mental fatigue moment in that condition. The increase in MDrow from LowF to HighF—Monotonous reflects the expected time-on-task effect under a constant cognitive load. The further increases observed in the demanding and dual-task conditions confirm the index’s responsiveness under more cognitively demanding scenarios. While both conditions always followed the monotonous task, the randomized order of administration helps rule out sequencing effects. Importantly, no statistically significant difference was observed between the HighF—Demanding and HighF—Dual-Task conditions. This is especially relevant when compared to the DALI scores, which indicated that the dual-task condition was perceived as more demanding. The lack of a corresponding MDrow increase suggests that the index does not simply reflect momentary cognitive workload, but instead tracks the progressive accumulation of mental fatigue. Asterisks (*) indicate statistically significant differences between the distributions connected by the corresponding brackets, as revealed by post hoc comparisons following repeated-measures ANOVA. * indicates *p* < 0.05 and ** indicates *p* < 0.01. Post hoc analysis using the Holm correction revealed significant differences between LowF and all other conditions (*p* < 0.001), indicating that the index sensitive to both passive mental fatigue (HighF—Monotonous) and active mental fatigue (HighF—Demanding and HighF—Dual-Task) while maintaining reliability and stability in the presence of additional cognitive demands (HighF—Demanding vs. HighF—Dual-Task).

Subsequently, the Friedman test was used to compare LowF, HighF—Monotonous, HighF—Demanding, and HighF—Dual-Task, yielding a significant result (χ^2^ = 29.118, *p* < 0.001, η^2^ = 0.511) (Figure 5).

**Figure 6:**
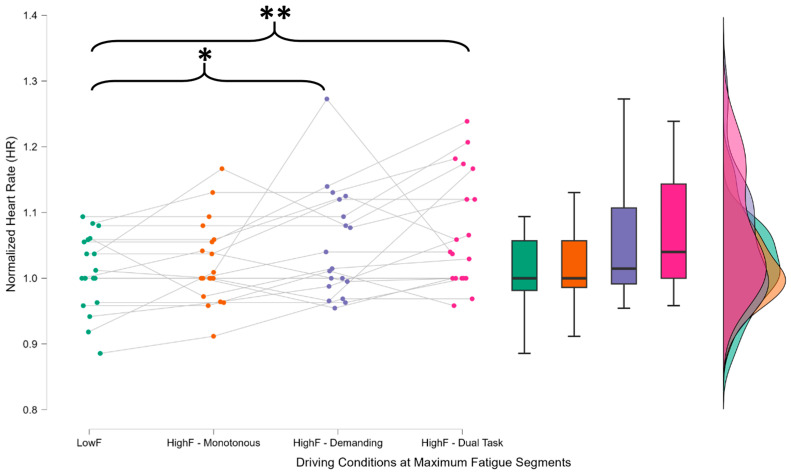
Normalized heart rate (HR) for each participant using their baseline recorded during the eyes-closed condition, computed during peak mental fatigue windows across the three driving tasks. The statistical analysis revealed a significant increase in HR during the demanding (HighF—Demanding) and dual-task (HighF—Dual Task) conditions, while no significant difference was found between LowF and HighF—Monotonous. This suggests that HR is more responsive to task-related cognitive load rather than to passive mental fatigue alone. The asterisks (*) indicate statistically significant differences between the distributions connected by the corresponding brackets, as identified through post hoc comparisons following repeated-measures ANOVA. * indicates *p* < 0.05 and ** indicates *p* < 0.01. The Friedman test revealed a significant effect for HRV (χ^2^ = 18.356, *p* < 0.001, η^2^ = 0.269), with a significant difference only between LowF and HighF—Demanding (*p* = 0.050) and no significant difference between LowF and HighF—Monotonous.

Regarding PPG data, ANOVA revealed a significant task effect for HR (χ^2^ = 15.348, *p* = 0.002, η^2^ = 0.269). Post hoc analysis indicated significance in the comparisons between LowF and HighF—Demanding (*p* = 0.002) and between HighF—Monotonous and HighF—Dual-Task (*p* = 0.018) (Figure 6).

**Figure 7:**
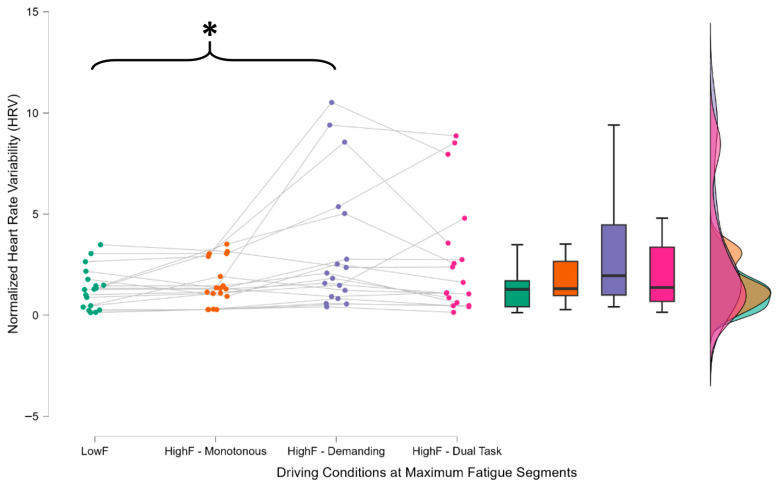
Normalized heart rate variability (HRV) for each participant using their baseline recorded during the eyes-closed condition, computed during peak mental fatigue windows across the three driving tasks. The statistical analysis revealed a significant increase in HRV during the demanding condition (HighF—Demanding), while no significant difference was found between LowF and HighF—Monotonous conditions. The asterisk (*) indicate statistically significant differences between the distributions connected by the corresponding brackets, as identified through post hoc comparisons following repeated-measures ANOVA. * indicates *p* < 0.05.

The lack of significance between LowF and HighF—Monotonous in both cases suggests that the variations are more closely related to the cognitive load of the task than to accumulated mental fatigue itself (Figure 7).

**Figure 8:**
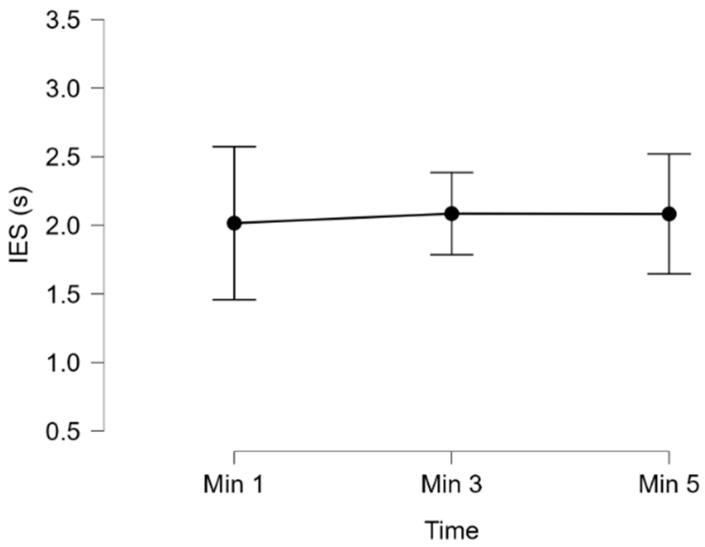
Performance in the secondary N-back task during the dual-task driving condition, expressed through the Inverse Efficiency Score (IES). The results revealed no significant effect of time, confirming that participants remained consistently engaged across the entire task. The error bars in the descriptive plot further support this finding, showing overlapping confidence intervals across the three timepoints.

Finally, an analysis of electrodermal activity (SCL and SCR) revealed no significant effects (SCL: F = 2.741, *p* = 0.077; SCR: χ^2^ = 3.169, *p* = 0.366, η^2^ = 0.056), indicating these measures were insensitive to changes in mental fatigue across different driving scenarios (Figure 8).

## 4. Discussion

This study aimed to evaluate the effectiveness of the MDrow index in detecting signs of active mental fatigue in cognitively demanding driving scenarios, extending its previous validation in monotonous driving conditions.

The MDrow index is based on the detection of Alpha spindles, brief bursts of activity in the lower Alpha band, typically found in central and parietal regions, which have been strongly associated with behavioral markers of mental fatigue, such as driving errors and increased blink duration in low-demand tasks [48]. Compared to traditional spectral power analysis, these transient events have shown greater sensitivity and specificity in detecting mental fatigue, even in ecologically valid driving conditions. The use of MDrow is supported by robust evidence highlighting the role of parietal Alpha synchronization, particularly through Alpha spindle, as a reliable marker of the onset and progression of mental fatigue. In resting states or during prolonged but low-demand tasks, a progressive increase in parietal and occipital Alpha activity has been documented, often accompanied by increased theta power and decreased beta activity [48,49]. In particular, rising lower-Alpha power has been correlated with subjective mental fatigue levels, indicating growing difficulty in sustaining alertness [49]. However, this neurophysiological profile may change under high cognitive load, as typically occurs in real-world multitasking scenarios. It is well known that Alpha activity is suppressed during cognitively engaging tasks [50]. Several studies have reported Alpha desynchronization, particularly in fronto-central and parietal regions, coupled with increased frontal theta activity, in response to elevated attentional and executive demands [48]. This pattern has been observed across a variety of cognitively demanding contexts, including flight simulation, air traffic control, working memory tasks, and multitasking driving [48]. Under these conditions, bilateral Alpha desynchronization can involve large portions of the parietal, central, and occipital cortex, reflecting increased cortical activation rather than decreased vigilance [48]. This raises the key question of whether EEG markers developed in monotonous driving contexts, such as MDrow, remain sensitive in more dynamic and cognitively complex scenarios, where mental fatigue is accompanied by elevated engagement. To address this, we employed a low-cost computational method with, at most, two accessory devices, using the MDrow index manually extracted from the EEG signal. In recent years, several approaches based on large-scale datasets and machine learning have been proposed for mental fatigue detection, including advanced architectures such as Convolutional Neural Networks (CNNs), which automatically extract temporal and spectral features from EEG [51,52], and Graph Convolutional Networks (GCNs), which capture functional relationships between brain regions [51]. While such models show great promise in terms of accuracy and generalizability, they rely on complex data-driven pipelines and multimodal integration strategies (e.g., EEG combined with eye-tracking or EOG [52]). In contrast, our study deliberately adopted a classical approach based on manual feature extraction, using the MDrow index, in order to evaluate whether this marker—originally developed and validated in monotonous driving conditions—retains its sensitivity under high cognitive load. This strategy allows us to directly test the robustness of MDrow in distinguishing different types of mental fatigue in more ecologically valid driving scenarios.

To this end, we designed a controlled experimental protocol including three simulated driving conditions (monotonous, demanding, and dual-task), combined with subjective and neurophysiological measurements. Subjective ratings (KSS and DALI) confirmed the effectiveness of the protocol: KSS scores showed a progressive and significant increase in perceived sleepiness (consistent with time-on-task effects), while DALI scores distinguished between the conditions, validating the contrast between passive (monotonous) and active (demanding/dual-task) mental fatigue. From a neurophysiological perspective, MDrow proved to be highly sensitive to the development of mental fatigue across different driving scenarios. Comparisons between Low-Fatigue (LowF) and High-Fatigue (HighF) conditions further confirmed MDrow’s sensitivity to both passive (HighF—Monotonous) and active (HighF—Demanding and Dual-Task) fatigue. Under the monotonous condition, MDrow values showed a clear and progressive increase from the lowest- to the highest-fatigue segments, despite the cognitive load of the task remaining constant. This strongly supports the interpretation that the index reflects the accumulation of mental fatigue rather than changes in task complexity. Crucially, however, no statistically significant difference was found between the HighF values of the demanding and dual-task conditions. This finding is particularly striking because it contrasts with the DALI scores, which indicated that participants perceived the dual-task condition as significantly more demanding. Behavioral data reinforce this interpretation: performance in the secondary N-back task during the dual-task condition, measured through the Inverse Efficiency Score (IES), showed no significant effect of time, demonstrating that participants remained consistently engaged throughout the task. This validates that the dual-task condition indeed imposed a higher cognitive load compared to the demanding condition. However, the absence of a corresponding increase in MDrow highlights that the index is not driven by momentary workload but rather by the progressive accumulation of mental fatigue. Taken together, these results underscore MDrow’s strength as a neurophysiological marker capable of disentangling mental fatigue-related processes from transient fluctuations in task demand. Physiological results from heart rate (HR) and heart rate variability (HRV) offered a more nuanced picture. Both HR and HRV significantly increased during cognitively demanding tasks, peaking in the HighF—Demanding condition. This aligns with the existing literature, where elevated HR is consistently linked to greater mental workload and increased sympathetic activation [48]. HR and HRV are commonly used indicators of autonomic response to cognitive stress, particularly in high-responsibility contexts such as aviation or simulated driving [48]. However, their link to mental fatigue appears less straightforward. During low-demand tasks, such as monotonous or night driving, HR has been shown to gradually decrease, reflecting reduced arousal and physiological slowdown. This suggests a biphasic response: HR and HRV initially rise with cognitive load but may decrease during cognitive resource depletion and the onset of drowsiness.

Accordingly, the absence of significant differences between LowF and HighF—Monotonous conditions supports the idea that HR and HRV primarily reflect mental engagement rather than accumulated mental fatigue, especially in the form of passive mental fatigue. These findings confirm that HR is more responsive to transient stress or effort, rather than to prolonged declines in vigilance [48]. In summary, while HR and HRV are useful markers of cognitive load during demanding tasks, they may lack sensitivity in detecting passive mental fatigue. This highlights the need to integrate them with other physiological signals, such as EEG biomarkers, for a more complete and accurate assessment of driver state. In contrast to EEG and cardiovascular measures, electrodermal activity (EDA), including skin conductance level (SCL) and skin conductance responses (SCR), did not show significant variations, appearing insensitive to both mental fatigue and task complexity in the present study. This finding partially contradicts the prior literature, which associates phasic EDA components, particularly SCR frequency, with changes in physiological arousal due to cognitive load. Previous driving studies have found SCR metrics to vary reliably with task complexity, whether induced by primary tasks, secondary tasks, or road characteristics [53]. Comparative analyses also suggest that SCR metrics outperform tonic SCL measures in distinguishing between levels of cognitive load, supporting the idea that transient sympathetic responses (phasic) are more directly linked to momentary mental effort. Neurophysiologically, SCL is associated with ventromedial prefrontal and orbitofrontal activity, while non-specific SCRs correlate with dorsolateral prefrontal cortex, anterior cingulate, bilateral insula, and cerebellum, regions more involved in cognitive processing [53]. Therefore, SCRs should, in theory, be more responsive to cognitive load. The lack of significant results in this study might be due to methodological issues (e.g., signal extraction), sympathetic saturation under prolonged stress, or the emotionally neutral nature of the cognitive tasks. Moreover, EDA is known to be affected by individual and environmental factors (e.g., temperature, sweating), which may have masked expected effects. These limitations suggest caution when relying solely on EDA to monitor mental fatigue and workload in realistic driving environments.

Overall, the findings reported support the adoption of the MDrow as a reliable EEG-based marker of mental fatigue in cognitively complex and realistic driving scenarios. Its robustness across varying levels of workload supports its potential future application in real-world research settings. However, some limitations must be acknowledged. The present experimental protocol was intentionally designed to validate the MDrow index under conditions of moderate mental fatigue, reflecting realistic driving scenarios rather than extreme states. Future studies will need to investigate the efficiency and accuracy of the index in detecting fatigue under more severe conditions, such as in sleep-deprived or heavily fatigued participants, in order to further strengthen its validation. In addition, to provide a more comprehensive picture of mental fatigue in the context of road safety, it will be important to take into account phenomena such as local sleep in wakefulness and microsleeps. Local sleep refers to brief, region-specific intrusions of sleep-like activity in the brain during wakefulness, while microsleeps are very short episodes of sleep that can occur even with eyes open. Both phenomena can significantly impair attention, reaction times, and overall driving performance, and therefore represent critical aspects to be considered in future research aimed at improving driver safety. Furthermore, although the participant sample was homogeneous, its size was not sufficient for subgroup analyses (e.g., by age or driving experience). Additionally, as controlled driving conditions were adopted, the simulated tasks do not fully replicate the unpredictability of real-world driving. Future studies should address these limitations by testing MDrow in on-road environments and integrating it with multiple physiological signals to develop a comprehensive multimodal mental fatigue assessment system.

## 5. Conclusions

This study confirmed the validity and sensitivity of the MDrow index for detecting both active and passive mental fatigue under conditions of moderate mental fatigue that are representative of realistic and recurrent scenarios typically experienced by drivers, through a simulated driving protocol. The results showed that the MDrow index reliably tracks mental fatigue progression, providing a continuous, objective, and robust measure even when additional cognitive activities are present.

The index proved sensitive to both passive and active mental fatigue, overcoming a major limitation of existing mental fatigue biomarkers, which are typically validated only under monotonous conditions. Furthermore, integrating the index with heart-related physiological parameters showed potential for a more comprehensive understanding of mental fatigue, though these parameters were more responsive to task-induced workload than to mental fatigue accumulation alone.

In summary, this study provides new evidence supporting the MDrow index as a valid indicator of mental fatigue applicable to realistic research settings. Its characteristics make it a promising tool for developing intelligent driver-monitoring systems, which could have implications for accident prevention and enhanced road safety.

## Figures and Tables

**Table 1:** Summary of the main features of the three driving scenarios. The first row presents the visual perspective of the driving scenario as displayed on the screen, corresponding to what participants saw during the task execution. The second row shows the map of the driving route to be followed by the participants. For the monotonous driving condition, the route corresponds to the default closed-loop layout of the scenario (a ring-shaped path). For the demanding and dual-task driving conditions, the routes were custom-designed to ensure that all participants performed the same number and type of maneuvers, and to include a variety of driving actions in order to enhance ecological validity. The third row displays representative images of the experimental setup, including the participant’s posture and the positioning of the recording devices during task execution.

	Monotonous Driving	Demanding Driving	Dual-Task Driving
Participants’ view	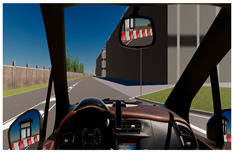	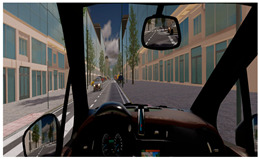	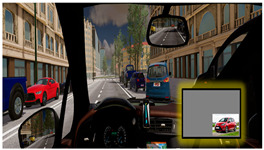
Driving route	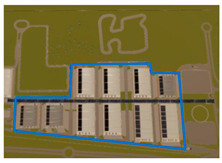	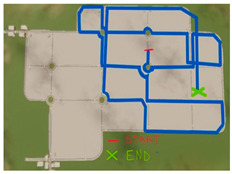	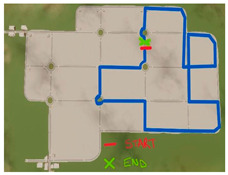
Setup overview	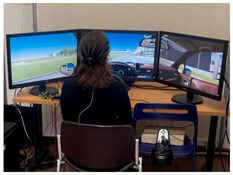	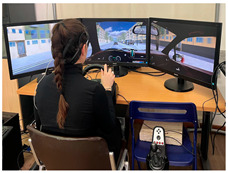	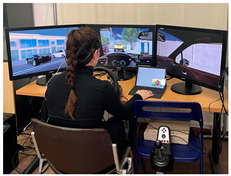

## Data Availability

The data presented in this study not are available on request present due to restrictions. They will be made publicly available after the completion of the funding project.
